# Clinical and molecular spectrum of patients with methylmalonic acidemia and homocysteinemia complicated by cardiovascular manifestations

**DOI:** 10.1186/s13023-025-03907-w

**Published:** 2025-08-19

**Authors:** Wanqing Zhao, Yanan Zhang, Yalei Pi, Yuqian Li, Huifeng Zhang

**Affiliations:** Department of Pediatrics, The Second Hospital of Hebei Medical University, Shijiazhuang, 050000 China

**Keywords:** MMA and homocysteinemia, MMACHC gene, Non-compaction of ventricular myocardium, Pulmonary hypertension

## Abstract

**Background:**

To investigate the clinical characteristics, treatment response, and prognosis of patients with methylmalonic acidemia (MMA) and homocysteinemia complicated by cardiovascular manifestations and to raise awareness regarding MMA and homocysteinemia.

**Methods:**

A total of 16 children diagnosed with MMA and homocysteinemia with cardiovascular manifestations who were admitted to the Department of Pediatrics of the Second Hospital of Hebei Medical University from June 2018 to October 2024 were retrospectively analyzed.

**Results:**

All 16 patients had varying degrees of neurological manifestations, and all had cardiovascular manifestations, 3 patients were diagnosed with MMA and homocysteinemia by newborn screening and received conventional treatment, the remaining 13 patients had nausea, vomiting, anemia, recurrent pneumonitis, respiratory distress, and lethargy as their first symptoms. Cardiovascular complications were found between the ages of 2 months and 12 years, with 9 patients having pulmonary hypertension, 7 having hypertension, and 5 having non-compaction of ventricular myocardium. Fourteen of these cases were confirmed to have CblC-type methylmalonic acidemia caused by mutations in the MMACHC gene by genetic testing. The most common mutations were c.80A > G (p.Q27R) (8 cases) and c.609G > A (p.W203X) (8 cases).

**Conclusion:**

Cardiovascular manifestation is uncommon in patients with MMA and homocysteinemia, but it is usually critical cause of death. When unexplained pulmonary hypertension or hypertension occurs, MMA and homocysteinemia should be suspected, especially when accompanied by manifestations of other systems.

## Background

Abnormal cobalamin metabolism within cells can lead to methylmalonic acidemia (MMA) and homocysteinemia [1]. Among these, the blc CblC type caused by MMACHC gene mutations is the most common form. Neurological impairment is the most common complication of MMA and homocysteinemia [2]. Cardiovascular manifestation is relatively rare in patients with MMA and homocysteinemia. This study has described the clinical manifestations, molecular spectrum, treatments, and prognosis of 16 children with MMA and homocysteinemia complicated with cardiovascular manifestation in order to improve the understanding of this disease.

## Method

### Object

Sixteen children with MMA and homocysteinemia complicated with cardiovascular manifestation who were admitted to the pediatric outpatient department of the Second Hospital of Hebei Medical University from June 2018 to October 2024 were included. The cohort comprised seven boys and nine girls. Informed consent was obtained from the children’s guardians and approved by the Clinical Research Ethics Committee of the Second Hospital of Hebei Medical University.

### Method

The data of 16 children were summarized: 1. Clinical history, encompassing gender, age, length, weight, age at diagnosis of MMA and homocysteinemia, clinical manifestations, diagnosis, therapeutic efficacy, etc. 2. Laboratory tests, including blood routine test, liver and kidney function, myocardial enzyme, electrolyte, blood gas analysis, BNP, homocysteine, etc. Blood amino acid and acylcarnitine profiles were determined by liquid chromatography–tandem mass spectrometry, urine organic acid analysis included urinary methylmalonic acid and methyl citric acid, and gene sequencing was performed using high-throughput sequencing and Sanger sequencing. 3. Imaging examination comprised cardiac ultrasound, cranial computed tomography, or magnetic resonance imaging, etc.

## Results

### Clinical data analysis

Among the 16 patients, three patients were diagnosed with MMA and homocysteinemia by neonatal screening and received regular treatment. Cardiovascular complications were found between 4 and 18 months of age. In the remaining 13 cases, cardiovascular complications were found at 2 months to 12 years, and they presented to the clinic with poor spirits, vomiting, cough, anemia, growth retardation, edema, recurrent pneumonia, shortness of breath, and lethargy as initial symptoms (Table 1).

**Table 1:** Analysis of clinical data of 16 cases

Case	Sex	Age	Time of Diagnosis MMA	First Symptoms	Cardiovascular System	Neurologic System	Hematologic System	Urinary System	Prognosis
1	Female	18 months	neonatal period	–	Pulmonary hypertension, Right heart enlargement, Tricuspid valve insufficiency,	Developmental delay	Orthocytotic anemia	–	Good
2	Male	4 months	4 months	Anemia, irritability	Pulmonary hypertension, Right heart enlargement, Pericardial effusion, Noncompaction of ventricular myocardium,Atrial septal defect	Developmental delay, old right frontotemporal hemorrhage	Orthocytotic anemia	Urinary occult blood, proteinuria	Good
3	Female	2 years	4 months	Growth retardation	Hypertension	Mental retardation, Developmental delay, dilatation of ventricular system	Macrocytic anemia	–	Good
4	Female	3 years	3 years	Vomiting, edema	Hypertension, Left heart enlargement	Developmental delay	Orthocytotic anemia, thrombocyto-penia	Urinary occult blood, proteinuria, Hemolytic uremic syndrome（HUS）	Die
5	Female	6 years	6 years	Vomiting, shortness of breath, edema	Hypertention, Left heart enlargement, non-compaction of ventricular myocardium	Mental retardation, developmental delay	Macrocytic anemia		Good
6	Male	12 years	12 years	Lethargy, cough,	Hypertension, Left heart enlargement	Developmental delay	–	IgM nephropathy	Good
7	Female	8 months	8 months	Anemia	Hypertension,Tricuspid valve insufficiency, Patent foramen ovale, Pericardial effusion	Mental retardation, developmental delay, widening of the external space between the bilateral frontotemporal brains	Orthoblastic anemia	Urine occult blood, proteinuria	Good
8	Female	7 months	neonatal period	–	Left heart enlargement, non-compaction of ventricular myocardium	Developmental delay	Orthoblastic anemia	–	Good
9	Male	6 years	6 years	Lethargy, chest tightness, cough	Right heart enlargement, Pulmonary hypertension, Ectopic left pulmonary vein introduced into coronary sinus,	Mental retardation, developmental delay	Macrocytic anemia		Die
10	Female	6 years	6 years	Anemia, edema	Pulmonary hypertension, Right heart enlargement, Tricuspidregurgitation	Developmental delay	Macrocytic anemia, thrombocyto-penia	Hemolytic uremic syndrome (HUS)	Die
11	Female	10 years	10 years	Cough, chest tightness	Hypertension, Pulmonary hypertention, Heart enlargement, non-compaction of ventricular myocardium	Developmental delay	–	–	Die
12	Female	4 months	neonatal period	–	Patent foramen ovale, Thickened left ventricular papillary muscle	Mental retardation, developmental delay, epilepsy, hydrocephalus	–	–	Good
13	Male	3 months	3 months	Shortness of breath	Patent foramen ovale, Pulmonary hypertension, Right heart enlargement	Developmental delay, epilepsy, hydrocephalus	–	–	Good
14	Male	6 years	6 years	Recurrent pneumonia	Enlarged heart, Pulmonary hypertension	Developmental delay	–	Urine occult blood	Good
15	Male	2 months	2 months	Anemia	Hypertension, Pulmonary hypertention, Left ventricular wall thickening, non-compaction of ventricular myocardium, Atrial septal defect, Patent foramen ovale, Pericardial effusion	Developmental delay, widening of anterior horns of the bilateral ventricles, widening of the extracerebral frontal lobe and longitudinal fissure of the brain, and widening of the cerebral sulci	Orthocytotic anemia, thrombocyto-penia	Urine occult blood, proteinuria	Die
16	Male	11 years	11 years	Shortness of breath	Pulmonary hypertension, Right heart enlargement, Tricuspid valve insufficiency	Developmental delay, demyelination lesions	–	–	Good

In our study, ten children showed different degrees of anemia, among which three of them were accompanied by thrombocytopenia. Five patients had abnormal liver function. Eleven children involved hematuria, proteinuria, and renal insufficiency. Case 6 was misdiagnosed with IgM nephropathy due to urinary protein, and case 4 and case 10 were diagnosed as hemolytic uremic syndrome (Table 2). All patients had varying degrees of developmental delay, and two of them had epilepsy. Some of them failed to complete the cranial imaging examination due to the rapid progression of the disease. Seven patients had complete cranial MRI examinations showing abnormalities including hydrocephalus, ventricular enlargement and/or extracranial space enlargement, demyelinating lesions, and frontal and temporal lobe hemorrhage (Table 1). Methylmalonic acidemia in urine and serum homocysteine was increased in all cases, and they all met diagnostic criteria for the MMA and homocysteinemia (Table 3).

**Table 2:** Results of routine hematuria and blood biochemical tests in 16 cases

Case	Hb(Female:110–150,Male:120–160)(g/L)	MCV(80–100)	PLT(100–300*10^9^/L)	ALT(9.0–50.0)(U/L)	AST(15.0–40.0)(U/L)	Cr(41–73)(umol/L)	Urine occult blood (–)	Urine protein(–)
1	107	94.3	299	10.5	31.7	26	–	–
2	49	84.1	159	14	32.3	47	2 +	3 +
3	67	109.5	152	9.9	30.6	34	–	–
4	63	97.1	57	10.7	37	162	3 +	3 +
5	74	107.1	255	9.8	31.9	65	2 +	3 +
6	118	95.7	268	9.7	27.8	149	–	–
7	70	85.3	333	11	34	28	3 +	2 +
8	60.6	96.9	325	50.8	32.1	143	–	–
9	100	101.2	153	102	204	414	–	–
10	98	112.7	82	818	683	703.5	–	–
11	119	79.5	200	65.6	52.9	276	–	–
12	115	95	220	24	28	18	–	–
13	112	97.6	315	35	29	32	–	–
14	116	97	323	134	120	23	2 +	–
15	66	88.7	72	17.2	33.5	29	3 +	2 +
16	134	90	120	9	19	31	–	–

*Hb* hemoglobin, *MCV* mean corpuscular volume, *PLT* platelet, *ALT* alanine aminotransferase, *AST* aspartate aminotransferase

**Table 3:** Results of tandem mass spectrometry and urine organic acid detection in 16 cases

Case	c0(10.00–100.00)	c2(4.50–65.00)	c3(0.30–5.00)	c3/c2(0.02–0.20)	Met(8.00–50.00)	mma(0.00–4.00)	Methyl citrate(0.00–0.80)	Hcy(0.00–15.00)(umol/L)
1	645.46	208.21	10.62	0.05	26.2	11.4	0.9	31.4
2	18.07	9.89	2.02	0.2	4.39	16.4	1.4	214.8
3	NA	NA	NA	NA	NA	NA	NA	179.6
4	68.22	91.8	11.73	0.13	12.9	53.9	2.7	31.7
5	51.11	38.54	8.87	0.23	11.21	20.5	0.4	212.9
6	39.22	21.93	13.7	0.63	18.78	44.3	0.5	136.6
7	360.79	22.04	8.22	0.37	9.04	12.7	1.2	334.23
8	NA	NA	7.6	0.32	NA	47.5	NA	49.1
9	NA	NA	10.2	0.31	NA	51.4	NA	54.2
10	NA	NA	15.2	0.36	NA	49.3	NA	63.3
11	94.22	114.81	13.32	0.12	11.96	69.5	1.0	194.1
12	82.15	20.2	14.8	0.73	16.6	602.6	0.8	55
13	18.7	15.78	9.44	0.6	NA	53.9	NA	103
14	NA	NA	3.25	0.21	NA	64.7	NA	136
15	8.14	4.55	4.74	1.04	3.52	192.3	8.3	149.4
16	13.70	10.77	6.78	0.63	22.10	510.7	7.1	97.3

*MMA* methylmalonic acidemia, *HCY* homocysteine

Among all 16 patients, 9 had pulmonary hypertension, 7 had hypertension, 5 had non-compaction of ventricular myocardium, 4 had patent foramen ovale, 2 had atrial septal defect, and 1 had anomalous entry of the left pulmonary vein into the coronary sinus. Right heart enlargement was found in 8 patients with pulmonary hypertension. Left ventricular enlargement was found in 5 patients with hypertension and /or  non-compaction of ventricular myocardium. In addition, there were 3 patients who only showed hypertension without left heart involvement. One patient with global cardiomegaly presented with pulmonary hypertension, hypertension, and non-compaction of ventricular myocardium (Table 4).

**Table 4:** Results of BNP and echocardiography detection in 16 cases

Case	LA(mm)	LV(mm)	RA(mm)	RV(mm)	EF(%)	CO	PAP(mmHg)	NT-pro BNP(pg/ml)
1	12	22	28	28	75	1.69	71	13,241
2	17	24	18	19	63.3	1.6	76	> 40,000
3	16	28	19	9	69.7	2.54	–	1970
4	19	39	25	13	54	3.81	–	> 35,000
5	24	51	28	12	46.72	8.89	–	> 35,000
6	35	58	38	18	59.41	11.16	–	> 35,000
7	18	26	23	11	78.9	2.19	–	15,498
8	14	32	17	10	51.28	2.56	–	233
9	15	25	48	25	71.43	1.88	95	> 15,000
10	16	33	50	27	69.5	3	95	> 15,000
11	30	46	33	30	29.2	2.76	50	> 40,000
12	19	23	7	17	69	2.34	–	NA
13	14	25	29	13	67	1.8	82	NA
14	18	45	32	16	54	2.45	97	NA
15	18	25	19	19	58.5	2.64	62	18,376
16	29	38	37	45	74.2	6.03	101	–

*LA* left atrium, *RA* right atrium, *LV* left ventricle, *RV* right ventricle, *EF* ejection fraction, *CO* cardiar output, *PAP* pulmonary artery pressure. *BNP* B-type natriuretic peptide

### Genetic testing

Of the 16 patients 14 were genetically tested. All confirmed to have pathogenic MMACHC gene mutations, including 3 homozygotes and 11 compound heterozygotes. Among these patients, the most common variants were c.80A > G (p.Q27R) (8 cases) and c.609G > A (p.W203X) (8 cases), of which two patients were homozygous for c.80A > G and one was homozygous for c.609G > A (Table 5).

**Table 5:** Results of genetic testing of the cases

Case	Gene	Nucleotide substitution	Amino acid substiotution	Exon position	Parenatal Original
1	MMACHC	c.80A > G c.80A > G	p.Gln27Arg p.Gln27Arg	Exon 1 Exon 1	Paternal Maternal
2	MMACHC	c.615C > A c.80A > G	p.Tyr205Ter p.Gln27Arg	Exon 4 Exon 1	Paternal Maternal
3	MMACHC	c.609G > A c.277-3_303del	p.Trp203* –	Exon 4 Exon3	Paternal Maternal
4	MMACHC	c.609G > A c.80A > G	p.Trp203* p.Gln27Arg	Exon 4 Exon 1	Paternal Maternal
5	MMACHC	c.80A > G c.609G > A	p.Gln27Arg p.Trp203*	Exon 1 Exon 4	Paternal Maternal
6	MMACHC	c.609G > A c.80A > G	p.Trp203* p.Gln27Arg	Exon 4 Exon 1	Paternal Maternal
7	MMACHC	c.658_660delAAG c.80A > G	p.K220del p.Gln27Arg	Exon 1 Exon 4	Paternal Maternal
8	MMACHC	c.656_658delAGA c.599G > A	p.Lys220del p.W200X	Exon 4 Exon 4	Paternal Maternal
9	–	–	–	–	–
10	–	–	–	–	–
11	MMACHC	c.80A > G c.80A > G	p.Gln27Arg p.Gln27Arg	Exon 1 Exon 1	Paternal Maternal
12	MMACHC	c.609G > A c.658_660delAAG	p.Trp203*	Exon 4 Exon 4	Paternal Maternal
13	MMACHC	c.609G > A c.609G > A	p.Trp203* p.Trp203*	Exon 4 Exon 4	Paternal Maternal
14	MMACHC	c.609G > A c.80A > G	p.Trp203* p.Gln27Arg	Exon 4 Exon 1	Paternal Maternal
15	MMACHC	c.658_660delAAG c.609G > A	p.K220del p.Trp203*	Exon 4 Exon 4	Paternal Maternal
16	MMACHC	c.482G > A c.656_658delAGA-	p.Arg161Gln p.Lys220del	Exon 4 Exon 4	Paternal Maternal

### Treatment and prognosis

After diagnosis of MMA and homocysteinemia, all patients were treated with vitamin B12, folate, levocarnitine, and betaine. Although we had taken active symptomatic treatment, five patients had a sharp deterioration after admission, which was difficult to reverse, and eventually died of heart failure. Patients 1, 2, 13, and 14 demonstrated normalization of pulmonary artery pressure and cardiac function following 6 to 12 months of treatment with cardiotonic drugs, sildenafil, and bosentan, prior to discontinuing the relevant medication. Case 16 had just started treatment with sildenafil, bosentan and digoxin and required further follow-up. In patients with hypertension, case 5 and case 6 were treated with cardiotonic drugs and captopril, and case 7 was treated with fosinopril. After 6 to 12 months of treatment, their blood pressure was stable and heart function improved.

## Discussion

Abnormal cobalamin metabolism within cells can lead to MMA and homocysteinemia [3]^.^ Among these, the cblc type caused by MMACHC gene mutations is the most common form. Significant individual differences among patients may result in damage to multiple systems throughout the body, including the nervous system, hematopoietic system, digestive system, kidneys, cardiovascular system, respiratory system [4]. Neurological impairment is the most common complication, with most patients exhibiting intellectual disability, developmental delays, epilepsy, and other symptoms [5]. Cardiovascular complications of MMA and homocysteinemia are not common but are usually critical and often the important causes of death [6].

Cardiovascular system involvement in patients with MMA and homocysteinemia can manifest at any stage of the disease [7]. In our study, the onset of cardiovascular complications in these patients ranged from 2 months to 12 years of age. Of these patients, 13 were not identified by neonatal screening and did not receive regular treatment in the early stage. Most of them showed severe cardiovascular complications at the onset of diagnosis. Therefore, untreated treatment may be a high-risk factor for cardiovascular complications in MMA and homocysteinemia.

Numerous studies suggest that cardiovascular complications in methylmalonic acidemia often present as pulmonary hypertension, hypertension, cardiomyopathy, and cardiac structural abnormalities, with pulmonary hypertension being the most common [8]. Pulmonary hypertension in MMA and homocysteinemia can be attributed to a variety of mechanisms and may be related to the accumulation of toxic metabolites such as homocysteine and methylmalonic acid [9]^.^ During normal fetal development, homocysteine metabolism relies on the methionine remethylation pathway rather than the transsulfuration pathway [1]. In patients with MMA and homocysteinemia, methionine synthase activity is reduced due to deficiency of the cofactor CblC, resulting in significantly elevated homocysteine levels in utero [10]. Elevated homocysteine induces the production of superoxide and peroxides, causing toxic damage to the vasodilatory function of endothelial cells [11, 12]. In addition, hyperhomocysteinemia induces the expression of coagulation factors, inhibits fibrinolytic function, prolongs thrombosis, increases thromboxane A2 production, and leads to vasoconstriction [11]. In our study, four out of the nine patients with pulmonary hypertension experienced rapid disease progression despite active treatment after admission, ultimately succumbing to irreversible heart failure. However, the remaining patients responded well to timely and effective treatment for the primary disease and received targeted drug therapy against heart failure and pulmonary hypertension. After 6 to 12 months of treatment, their heart function significantly improved and pulmonary hypertension was alleviated, with no recurrence observed during follow-up.

Of the 7 patients with hypertension, 3 patients died of heart failure (2 of whom also had pulmonary hypertension), and the heart function of the other patients returned to normal after blood pressure was controlled. Prior literature suggests that patients with MMA and homocysteinemia and hypertension often exhibit renal involvement, indicating that renal lesions may contribute significantly to hypertension in these patients. However, the findings in this study, where three out of seven patients with hypertension did not display renal involvement, suggest that factors other than renal lesions may also play a role in the development of hypertension [13]. For example, elevated homocysteine, an independent risk factor for cardiovascular disease, may contribute significantly to the development of hypertension [14].

Our study suggested that cardiac enlargement in patients with MMA and homocysteinemia was mostly secondary to pulmonary hypertension and/or hypertension, and only one case is secondary to non-compaction of ventricular myocardium. For patients with MMA and homocysteinemia, if pulmonary hypertension and/or hypertension can be detected and corrected in time, the prognosis can be significantly improved. At the same time, our study showed that non-compaction of ventricular myocardium was more common in patients with MMA and homocysteinemia than dilated cardiomyopathy. Left ventricular non-compaction is characterized by abnormal trabeculations in the left ventricle, most frequently at the apex. It can be associated with left ventricular dilation or hypertrophy, systolic or diastolic dysfunction, or both, or various forms of congenital heart disease. Affected individuals are at risk of left or right ventricular failure, or both. Genetic inheritance arises in at least 30–50% of patients, and several genes that cause left ventricular non-compaction have been identified. These genes seem generally to encode sarcomeric (contractile apparatus) or cytoskeletal proteins, although, in the case of left ventricular non-compaction with congenital heart disease, disturbance of the NOTCH signaling pathway seems part of a final common pathway for this form of the disease. More studies are needed to clarify the relationship between MMA and homocysteinemia and non-compaction of ventricular myocardium, as well as its prognosis.

In our study, 2 patients failed to refine their genetic testing after the rapid progression of their disease after onset. Another 14 patients had mutations in the MMACHC gene confirmed by genetic testing. It is located on autosomal lp34 and is responsible for encoding the CblC protein, which catalyzes the reduction and decytosis reactions of CNCbl, as well as the synthesis of adenosylcobalamin and methylcobalamin in the cytoplasm [15, 16]. A total of 9 mutant sites were detected in the 14 patients, with the high-frequency variant sites being c.80A > G (8 patients) and c.609G > A (8 patients). These two sites are hotspot mutations in the MMACHC gene in the Chinese population [17], and may also be hotspot mutations for cardiovascular complications in MMA and homocysteinemia [18].

## Conclusions

Cardiovascular involvement is relatively rare in methylmalonic acidemia combined with homocysteinemia. Early detection and timely control of cardiovascular complications can significantly improve outcomes [19]. Therefore, clinicians must be vigilant about cardiovascular complications during diagnosis and treatment. When unexplained pulmonary arterial hypertension or hypertension occurs, the presence of methylmalonic acidemia and homocysteinemia should be suspected [13].

## Data Availability

The datasets used and/or analysed during the current study are available from the corresponding author on reasonable request.

## Abbreviations

MMA: Methylmalonic acidemia
HCY: Homocysteine
Hb: Hemoglobin
MCV: Mean corpuscular volume
PLT: Platelet
ALT: Alanine aminotransferase
AST: Aspartate aminotransferase
LA: Left atrium
RA: Right atrium
LV: Left ventricle
RV: Right ventricle
EF: Ejection fraction
CO: Cardiar output
PAP: Pulmonary artery pressure
BNP: B-type natriuretic peptide
